# Ovarian recovery via autologous platelet‐rich plasma: New benchmarks for condensed cytokine applications to reverse reproductive aging

**DOI:** 10.1002/agm2.12196

**Published:** 2022-02-05

**Authors:** E. Scott Sills

**Affiliations:** ^1^ FertiGen CAG/Regenerative Biology Group San Clemente California USA; ^2^ Department of Obstetrics & Gynecology Palomar Medical Center Escondido California USA

**Keywords:** fertility, menopause, ovary, PRP, women's health

## Abstract

Health and life expectancy gains have pushed the overall number of menopausal patients to record levels. Because maternal age at first pregnancy also continues to rise, it is unsurprising that reduced birth rates are consistently reported across many populations. Both trends severely strain national demographics and present a socioeconomic challenge for which no satisfactory solution currently exists. Symptomatic menopause and infertility/miscarriage are met with standard therapies like hormone replacement therapy (HRT) and in vitro fertilization, respectively. Although these accepted interventions do supply some cover, both are expensive, low yield, and not without controversy. Meanwhile, ovarian steroid output and competent oocyte availability approach unrecoverable loss beyond age ~35 years, irrespective of treatment. Received wisdom holds that postnatal oogenesis in humans is impossible, a tenet which until recently encountered little serious confrontation. Reassessing this paradigm is overdue given proof‐of‐concept work on native sex steroid rejuvenation, de novo euploid oogenesis, ovulation, blastocyst development, fetal growth, and healthy term livebirths—all apparently possible with intraovarian insertion of platelet‐rich plasma (PRP). Discrete functional analysis of the full platelet‐derived cytokine array carried with PRP unfortunately for now, is incomplete. Here, selected platelet releasate constituents and measured effects are framed to address advances in wellness and women’s health. Emphasis is on cytokines best positioned to enable recovery of senescent ovarian function sufficient to suspend synthetic HRT dependency and/or permit egg retrieval and pregnancy. Whereas the chronicle of progress in other clinical fields does invite generalization of fresh platelet applications to reproductive endocrinology, basic mechanistic questions remain open.

## Graphical abstract

Representative technique for intraovarian insertion of platelet‐rich plasma (PRP) or enriched plasma cytokines. A sample from peripheral venipuncture (a) is processed to separate and activate fresh autologous platelets (b), which may be further incubated to yield enriched platelet factors (EPFs) as a cell‐free condensate (b′). Relevant mediators in either PLT releasate preparation include platelet factor 4 (PF4) involved in PLT aggregation; interleukin‐1β (IL‐1β) a central inflammatory signal directing cell proliferation, differentiation, and apoptosis; and IL‐8, which coordinates angiogenesis. Under direct ultrasound guidance, either activated PRP or EPF is injected into ovarian tissue via 19G single‐lumen in vitro fertilization needle. Periodic assessment of serum anti‐Mullerian hormone provides documentation of ovarian reserve response after treatment, including return of menses and follicular development.

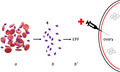

## 1 INTRODUCTION

Although advanced fertility treatments have been available since the early 1980s, this technology is still largely inaccessible or unaffordable in many countries. Even in high‐income nations, how curative resources should be equitably offered as value‐for‐money has been questioned.^1^, ^2^ Until 2010, the US National Assisted Reproductive Technology Surveillance System posted steady gains in livebirths using the most costly and sophisticated treatment available—in vitro fertilization (IVF). However, for unclear reasons, by 2016, outcomes from IVF had fallen to levels not seen since the mid‐1990s.^3^ Median female IVF age held stable at 36 years during this period, aligning with the tendency to refer older IVF patients into oocyte donor programs where they are tabulated separately.^3^ With menopause care, treatment goals have similarly unraveled as real‐world hormone replacement therapy (HRT) discontinuation in this group occurs far more often than officially reported.^4^ Insufficient data exist to guide use of one HRT method or dose over another,^5^ which may explain why three quarters of menopausal women stop taking prescribed hormones before any beneficial effect is achieved.^4^ Because infertility and menopause are both captive to ovarian reserve, developing a safe and effective recovery of ovarian function—perhaps without synthetic pharmaceuticals—would supply a shared answer for two eminent women’s health questions.

## 2 CURRENT PROBLEM

Producing hormones necessary for puberty and later to sustain egg/embryo development, the ovary is the master fertility regulator throughout the female reproductive career.^6^ Competent (genetically balanced) oocyte development is the sum of many cellular, environmental, and nutritional milestones cleared long before ovulation. If successful, this allows production of one healthy egg and only later will a derivative unique embryonic genome be activated.^7^ Given this extended developmental prelude, when blastocyst aneuploidy affiliated to advanced maternal age is a clinical concern, any changes to IVF gonadotropin protocols enter too late to provide any meaningful effect. Accordingly, interventions to optimize embryonic chromosomal integrity must somehow adjust the maternal ovarian climate upstream, well ahead of folliculogenesis or oocyte collection.^8^ Reprogramming, differentiation, and recruitment concepts discussed here are distinct from IVF gonadotropin details during follicular recruitment/ovulation induction, and instead pivot on autologous platelet factor cross‐communication with ovarian stem cells.

**FIGURE 1:**
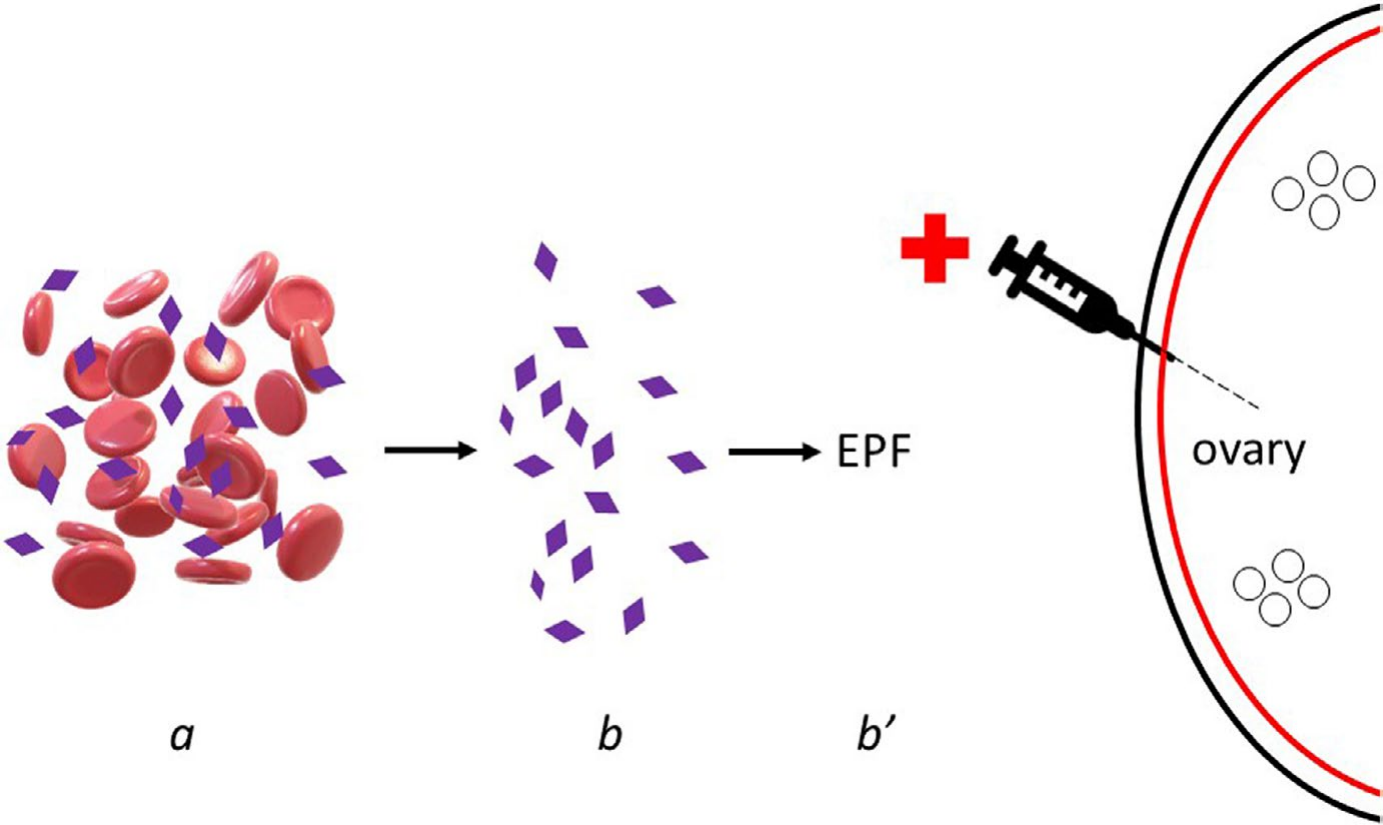
Representative technique for intraovarian insertion of platelet‐rich plasma (PRP) or enriched plasma cytokines. Sample from peripheral venipuncture (a) is processed to separate and activate fresh autologous platelets (b), which may be further incubated to yield enriched platelet factors (EPF) as a cell‐free condensate (b′). Relevant mediators in either PLT releasate preparation include platelet factor 4 (PF4) involved in PLT aggregation; interleukin‐1β (IL‐1β) a central inflammatory signal directing cell proliferation, differentiation, and apoptosis; and interleukin‐8 (IL‐8), which coordinates angiogenesis. Under direct ultrasound guidance, either activated PRP or EPF is injected into ovarian tissue via 19G single‐lumen in vitro fertilization needle. Periodic assessment of serum anti‐Mullerian hormone provides documentation of ovarian reserve response after treatment, including return of menses and follicular development

Several methods to place platelet‐derived cytokines into ovarian tissue can be undertaken (see Figure 1), although all aim to improve ovarian reserve (i.e., the functional capacity to ovulate competent oocytes and produce sex steroids, all of which decline with advancing age).^6^ For example, conventional “ovarian rejuvenation” involves surgical placement of autologous (activated) platelet‐rich plasma (PRP) into ovarian tissue, either by laparoscopy or transvaginal ultrasound‐guided injection. If platelets are cultured/isolated to permit collection of cytokines (“cargo proteins”) after in vitro release, then this cell‐free product may also be inserted for intraovarian use. At present, no head‐to‐head comparisons to assess such varied techniques exist.^9^ The first autologous ovarian PRP clinical trial^10^ enrolled > 150 poor‐prognosis women with a history of prior IVF failure, as a proof‐of‐concept measure. It tracked ovarian reserve response and observed significant anti‐Müllerian hormone (AMH) increases in > 25% of patients after intraovarian PRP. That ovarian injection itself was an independent causative factor for improved function was not validated by data, as baseline platelet concentration (not the surgical procedure) was correlated to serum AMH response.^10^ Comparable results have since been obtained from other fertility units of international provenance, with no adverse events reported.^9^

## 3 RECENT PROGRESS

The rejuvenation potential of platelet‐derived cytokines is difficult to overstate. In *Gekko japonicus*, a study of the platelet‐derived growth factor‐C (*PDGF‐C*) gene^11^ found strong and diffuse expression across cardiac, renal, pulmonary, and ovarian tissue. Intriguingly, expression of PDGF‐C in the spinal cord measured after tail amputation suggested that PDGF‐C is plausibly associated with spinal cord injury recovery and tail regeneration.^11^ Less dramatic tissue injury can upregulate transforming growth factor β (TGF‐β), followed by differentiation, extracellular matrix remodeling, and fibrosis. Although the molecular mechanisms of this differentiation and survival need additional study^12^ it is known that TGF‐β is a component of human platelet releasate.^6^

Oxidative stress in response to ovarian trauma or aging is normally held in check via signal transduction and redox regulation, but when excessive, the disruption becomes irreparable. Under normal conditions, a complex antioxidant protection system maintains the redox state across a variety of cells.^13^ However, as antioxidant defense levels decline with further aging, the balance between reactive oxygen species (ROS) production and clearance favors the former. ROS accumulation triggers autophagy, a lysosome‐mediated process required for protein and organelle quality control. Although younger eggs can offset oxidative injury to some extent, increased age brings gradual impairment in this correction. DNA repair is slowed and reduced effectiveness of the spindle assembly checkpoint occurs with decreased capacity for protein repair and degradation.^6^ Thus, any age‐related hypoxia or other ovarian stress further compromises the older egg’s already‐effete capacity to mitigate such damage. Introduction of platelet cytokines into this milieu has been experimentally shown to ameliorate tissue injury via inhibition of oxidative stress and inflammation.^14^ Because platelet cargo proteins remain incompletely inventoried, it remains uncertain which PRP component (or combination of components) is needed for this rescue.

Among the members of the platelet releasate ensemble is bone morphogenetic protein‐11 (BMP‐11), also termed “growth differentiation factor 11” (GDF). Tissue rejuvenation and cellular anti‐aging have long been associated with BMP‐11 in general, but its role in the ovary is only now receiving specific study. Zhou et al^15^ developed an orally bioavailable recombinant form of this mediator (rGDF‐11) for testing in mice, and subsequently observed increased tissue antioxidant enzymes, delayed ovarian aging, restoration of gonadal endocrine activity, and fertility improvement.^15^ Of note, this growth factor is highly concentrated in platelets and PRP.^16^, ^17^

Research consistently fixes the ovary as the key node within a multiplex signaling field, important in upregulating genes involved in adaptive and innate immunity. Such innate immune hyperactivity fits well with the chronic inflammation commonly accompanying increased age, especially prominent in aged ovaries.^18^ For example, flow cytometry revealed M2 polarization to be more common among macrophages with increasing age, whereas M1 polarization demonstrates marked decline. Curiously, similar macrophage changes have been reported in murine retinal tissue over time.^19^ Zhang et al have proposed that an altered macrophage plasticity, elevated type 2 immunity, and M2 macrophage action all converge to yield ovarian fibrosis.^18^ Considering this hypothesis, clinical interventions aiming to arrest or reverse such organ sclerosis by opposing mediators contributing to functional collapse warrant close notice. Furthermore, as macrophages appear to undergo proliferation in vivo with estrogen exposure,^20^ the paucity of this indispensable female hormone seen with advanced age gradually dims another promoter of macrophage action. Clinical trial data suggest this downward estrogen spiral can be rewound with intraovarian PRP,^21^ thus replenishment of the ovarian macrophage pool and blunting of inflammatory signaling may be a secondary effect of this approach.^22^

PRP is a known attenuator of inflammatory NF‐κB signaling.^23^ Western blot, real time polymerase chain reaction, and enzyme‐linked immunosorbent assay have documented apoptosis‐related signaling and inflammatory mediators in a mouse model, and confirmed that PRP blocked dysregulation of SOX9, Col2A1, Col10A1, and Aggrecan, lowered levels of inflammatory markers COX‐2 and iNOS, and reduced doxorubicin‐induced phosphorylation of IκB and NF‐κB.^23^

This tissue/organ finding in mice was an extension of prior data published by Iranian experts who used an experimental autograft design to assess ovarian tissue alone or with platelet‐rich fibrin (PRF).^24^ Their research found total ovary volume, follicle number, superoxide dismutase activity, total antioxidant capacity, IL‐10, progesterone, and estradiol were all significantly higher in the group, including PRF. Moreover, angiogenesis was accelerated, whereas apoptosis and malondialdehyde concentrations were significantly lower when platelet cytokines were present, compared to PRF‐free controls.^24^

Mesenchymal stem cells (MSCs) have been used experimentally in animals to correct undesirable aspects of ovarian aging. Mammalian MSCs generate a cytokine array which closely parallels that are produced by activated human platelets, including hepatocyte growth factor, vascular endothelial growth factor, and insulin‐like growth factor‐1. Such factors are known to boost ovarian function and resist/delay tissue senescence. Specifically, MSCs of umbilical cord^25^ and amniotic source^26^ can promote growth factor expression required for organogenesis, cellular homeostasis, and tissue regeneration.^27^ All these mediators are well‐represented within the cytokine releasate suite following platelet activation.^9^

Quantifying specific platelet‐derived cytokine elements, although certainly informative, is not guaranteed to unlock every mystery associated with PRP function. By example, changes in joint space cytokine levels and clinical response were measured in patients diagnosed with knee osteoarthritis receiving either two or four intra‐articular PRP injections. Irrespective of which PRP schedule was followed, paradoxically, there were no differences in synovial inflammatory cytokines, anti‐inflammatory cytokines, or growth factors. Nevertheless, both patient groups had better 6 week’s clinical outcomes for up to 1 year.^28^ The inability to associate laboratory data reliably with patient response foreshadows a long path ahead for PRP research.

## 4 PRACTICE APPLICATIONS

One method of intraovarian injection of PRP has been previously published,^13^ which outlines patient intake, baseline assessment, specimen processing, ovarian insertion, and post‐treatment evaluation to document response. In this equation, platelet activation is a first‐order term, as this facilitates vital platelet growth factor release.^6^ Different reagents and platelet incubation protocols exist, but to date consensus is lacking for which culture and condensing sequences are preferred. As expected, suboptimal laboratory practices are common reasons platelet releasate biopotency is diminished, thus culminating in ovarian nonresponse.^29^

Follicular oxygen saturation in the microclimate of the developing oocyte is now considered central in determining gamete competence. Inadequate follicular perfusion ends in oxidative stress and tissue injury, a circumstance which can only be partially influenced by exogenous gonadotropin parameters. In contrast, when administered perhaps 3 months before planned oocyte harvest, intraovarian injection of PRP and/or platelet‐derived growth factors (as a cell‐free substrate) appears to redress this dysfunction.^13^

During informed consent, safety concerns about platelet cytokines/PRP, especially the potential to induce tumorigenic changes after intraovarian injection, should be carefully discussed. As this outcome has never been observed for more than 10 years of clinical PRP use in any clinical context, it likely reflects only a theoretical risk. Platelet cytokines interact with cell membrane—not nuclear—receptors so the biological effect is distinct from trophic hormones.^30^ Experience with other tissues treated with PRP reduces doubt further, as recruitment of normal cells is achieved with no subsequent malignancy.^31^

## 5 CONCLUSION

Health and reproductive capacity are closely linked.^32^ Investment in women’s health promotion and wellness will need to assure that ovarian insufficiency and menopause are priorities among portfolio aims. For now, “ovarian rejuvenation” seems better suited to address infertility/miscarriage than for menopause treatment, as most patients would probably regard PRP as impractical and uncompetitive against HRT. Indeed, a cost projection for ovarian PRP and standard HRT^33^ underscored the crushing inefficiency of ovarian PRP when used to assuage symptomatic menopause. However, if plasma cytokine research can enable a targeted ovarian response more efficiently, the cost/benefit calculus might hold more appeal.

Reproductive impairment is common in mammals and is congruent with disease frequency, especially for women who are generally advantaged (until menopause) over men.^34^ It has been shown that surgical grafting of young ovarian tissue into post‐reproductive‐aged women improves survival significantly.^32^ However, as reproductive potential decays and vanishes, the incentive to preserve the somatic health of the organism appears to be lost as well.^34^ On balance, intraovarian insertion of platelet‐derived cytokines might offer a new, safe, and nonpharmacologic therapy for some women. This is a hopeful moment within the field of reproductive physiology, with experimental and clinical activity poised to offer important discoveries. Alternatives to accepted thinking must be encouraged, particularly where “final frontiers” may seem forbidding.^35^

## ACKNOWLEDGMENTS

None.

## CONFLICT OF INTEREST

The author has been awarded U.S. Trademark #88505430 for treatment of female hormone and fertility enhancement utilizing a specified method of intraovarian injection of autologous platelet rich plasma.
